# Barriers and Facilitators to Incorporating an Integrative Mind–Body Intervention in Youth With Type 2 Diabetes

**DOI:** 10.1016/j.jaacop.2024.01.005

**Published:** 2024-02-15

**Authors:** Irina Bransteter, Molly McVoy, David W. Miller, Rose A. Gubitosi-Klug, Tracy L. Segall, Mina K. Divan, Jessica Surdam, Martha Sajatovic, Jeffery A. Dusek

**Affiliations:** a Case Western Reserve University School of Medicine, Cleveland, Ohio; b University Hospitals Cleveland Medical Center, Cleveland, Ohio; c Rainbow Babies and Children, UHCMC, Cleveland, Ohio; d University Hospitals Connor Whole Health, Cleveland, Ohio; e University of California – Irvine, Irvine, California

**Keywords:** qualitative, diabetes, AYA, mind–body, self-management

## Abstract

**Objective:**

There has been little to no qualitative research done with adolescents and young adults (AYA) with type 2 diabetes (T2D) that can guide creation of interventions for this demographic. Using qualitative research methods, a novel mind–body intervention called Intervention for Early Onset Type 2 Diabetes (INTEND) has been developed for AYA aged 15 to 20 years, with the goal of improving self-management and coping skills, by enhancing routine care with augmented education coupled with mind–body skills.

**Method:**

Qualitative interviews with AYA 15 to 20 years of age with T2D, their parents, and professionals caring specifically for this population were done through a focus group model. Transcripts were created, depersonalized, and coded using a Consensual Qualitative Research (CQR) method. Identified themes then guided the creation of course materials that included education about self-management of T2D and how to use the 4 mind–body technique toward self-care and regulation.

**Results:**

The qualitative approach used in the development of this intervention revealed important findings in understanding key barriers faced by this group, key facilitators that improve their quality of life, and core components of an intervention that would be acceptable to them.

**Conclusion:**

Results of this qualitative study helped craft an intervention tool that can subsequently be deployed and evaluated for effectiveness. Findings of the qualitative research model allow us to better understand the lived experience of AYA living with T2D.

**Clinical guidance:**

• Stigma of type 2 diabetes in adolescents may interfere with patients’ ability to adequately adhere to treatment recommendations
• Clinicians need to identify social supports for adolescents with type 2 diabetes
• Identifying family members and including them in treatment plans may help adolescents with type 2 diabetes

## Plain language summary

Youth-onset type 2 diabetes is a growing epidemic associated with increased risk for mental health problems, but there is little research regarding tailored treatment options. This study examined data from key stakeholder input from 3 patients, 3 parents, and 6 healthcare professionals using focus groups and qualitative interviews, to identify themes that guided the development of a novel mind-body intervention. Adolescents, medical providers and guardians identified stigma, isolation, and difficulty coordinating medical care as barriers to care. The novel mind-body intervention addressing both physical and mental health was positively received by all stakeholders. Thess results indicate the acceptability of a novel treatment for this understudied and underserved population.

Diabetes mellitus is a medical condition defined by elevated blood glucose levels, as measured by fasting blood glucose (FBG) ≥126 mg/dL, 2-hour glucose during an oral glucose tolerance test (OGTT) ≥200 mg/dL, or hemoglobin A1c (HbA1c) ≥6.5%.^1^ Symptoms include increased thirst and urination. Type 1 diabetes (T1D) is the most common type in the pediatric population; it results from autoimmune destruction of the pancreatic islet β cells that leads to a deficiency of insulin, the hormone that reduces blood glucose levels. T1D can present in any decade of life, but onset is more common in children, with the incidence most rapidly increasing in children less than 6 years of age. In contrast, type 2 diabetes (T2D) is more common in adults and occurs in the setting of insulin resistance, largely related to excessive adiposity and leading to pancreatic dysfunction and, ultimately, pancreatic failure over time. With the rise in pediatric obesity, the incidence of youth (10-19 years of age) with pancreatic autoantibody–negative diabetes consistent with T2D is increasing.^2^^,^^3^ Similar to adults with T2D, youth-onset of T2D is more common among racial and ethnic minorities. Other known risk factors include a family history of T2D in a first- or second-degree relative, exposure to gestational diabetes, use of antipsychotic medications, and a history of intrauterine growth retardation or small for gestational age at birth. Any presentation of new-onset diabetes mellitus in a youth requires consideration of both types, as rarely patients presenting with excessive adiposity may also have autoantibodies; the latter heralds type 1 diabetes (T1D) and the requirement of management with insulin.

Youth-onset T2D is a growing epidemic,^4^ with significant associated cost and disease burden. There was a 7% annual increase in T2D rates in US youth between the years 2002 and 2012.^5^^,^^6^ As many as 5,000 new cases of T2D are diagnosed in US youth each year.^7^

T2D in youth produces earlier and more severe complications than experienced by youth with T1D or for individuals with adult-onset T2D,^8^ including elevated body mass index and blood pressure, worsened blood glucose control, and increased T2D illness duration.^9^^,^^10^

As demonstrated by the Treatment Options for Type 2 Diabetes in Adolescents and Youth (TODAY) study, diabetes comorbidities and complications present early and progress at an aggressive rate in youth diagnosed with T2D.^11^ This longitudinal, multicenter treatment trial followed participants from diabetes onset until their mid-20s with an average diabetes duration of 13.3 years. Comorbidities such as hypertension and dyslipidemia were present at diabetes onset in approximately 20% of youth; the cumulative incidence increased to about 30% within 5 years of diagnosis, and rose to 67.5% and 51.6%, respectively by study end. Microvascular complications including early diabetic retinopathy was found in 13% of participants at 5 years post-diagnosis, increasing to 51% experiencing non-proliferative diabetic retinopathy and 12% with more advanced retinal disease, including sight-threatening proliferative diabetic retinopathy and macular edema within just 7 additional years. Diabetic kidney disease was present in 8% of participants at baseline and in over half of participants by study end. Together with the challenge of reaching and maintaining glycemic targets, these comorbidities and evidence of microvascular disease raise concerns for an increased risk of future cardiovascular disease. Albeit rare, concerning was the occurrence of even a few cases of major adverse cardiovascular disease events, including myocardial infarction, coronary artery disease, congestive heart failure, and stroke in participants in their mid-20s at an average diabetes duration of just over a decade.

Adolescents and young adults (AYA) with T2D are at an increased risk for mental health problems, including poor sleep, depression, and anxiety.^12^^,^^13^ Mental health comorbidity, in turn, worsens risks for more physical health complications in youth with T2D. Adolescence, as a developmental time frame, holds its own unique challenges, and AYA with chronic health conditions must be approached in developmentally and culturally appropriate ways. Despite the substantial and increasing burden of youth-onset T2D, little is known regarding barriers and facilitators to treatment.^14^, ^15^, ^16^, ^17^, ^18^ There is very little research regarding AYA with T2D and mental health symptoms and complications.^7^^,^^13^^,^^19^ The Intervention for Early Onset Type 2 Diabetes (INTEND) study was undertaken to explore novel ways of understanding and augmenting this knowledge base. INTEND was developed for AYA 15 to 20 years of age and had 2 phases, a qualitative research component to inform the development of a group education program, addressing identified barriers and facilitators with augmented education and mind–body techniques. The intervention is not scoped to treat major psychiatric issues, and participants are expected to be under conventional care for cases that would benefit from medication. Although the intervention may not sufficiently provide full psychological care, it is expected to be supportive in this domain. Future research will include measures of anxiety, depression, and quality of life to assess level of improvement in participants.

There is growing evidence that mind–body and self-management interventions improve long-term outcomes, including improving glycemic control, lowering body mass index, improving lipid profiles, improving quality of life, and improving mental health.^20^, ^21^, ^22^, ^23^, ^24^, ^25^, ^26^ In adults, there is substantial evidence for mind–body practices and integrative care therapies for a broad range of chronic medical illnesses.^27^^,^^28^ In children and adolescents, there is also significant evidence for integrated care, especially in hospitalized youth, children with cancer, and children with chronic pain.^29^^,^^30^ There is a small but promising body of literature showing the potential benefits of mind–body techniques in T2D.^31^ Yang *et al.* discussed physiologic mechanisms by which mind–body interventions might have an impact on T2D, including altering cortisol levels, regulating the autonomic nervous system, and decreasing inflammation.^32^ A case report describes the impact of a mind–body intervention on outcomes for a 16-year-old participant, with increased mindfulness, decreased depressive symptoms, and improved insulin sensitivity immediately following the intervention and at 1 year.^33^ Improvements in cholesterol levels and moderated inflammatory gene expression were noted in patients with hypertension and/or T2D using a “brain education–based meditation” strategy.^34^ We found no instances, however, where a qualitative research design was used to help craft a mind–body augmented program specifically tailored to the needs of AYA with T2D while taking into account the psychoemotional needs of the population. Addressing this specific aspect of care with AYA, parent, and medical team input appears to be novel.

## Method

### Sample and Setting

Participants (N = 12) were recruited through outreach to the Pediatric Endocrinology, Integrative Medicine, and Behavioral Health Departments at University Hospitals Cleveland Medical Center in Cleveland, Ohio. The electronic health record was screened for T2D diagnosis, which was determined by the following: FBG >126 mg/dL; or 2-hour OGTT >200 mg/dL; or HbA1c ≥6.5%; or the presence of symptoms and random blood glucose >200 mg/dL. The sample included the following: (1) 3 female AYA participants, mean age 17.33 years (range, 16-19 years), diagnosed with T2D as well as comorbid depression and/or anxiety; (2) 3 parents of AYA diagnosed with T2D, as well as comorbid depression and/or anxiety, mean age 45 years (range, 39-49 years); and (3) 6 healthcare professionals, including a registered nurse, a social worker, a diabetes educator, a psychiatry fellow, an endocrinologist, and a dietician, with mean years of practice 17 years (range, 3-23 years). AYA ranged from 1.5 to 4 years since diabetes diagnosis, and all were diagnosed with both anxiety and depression. All 3 AYA are receiving standard psychiatric services, and 1 AYA is receiving therapy as well.

### Study Design and Qualitative Data Collection and Analysis

The study was approved by the University Hospitals Institutional Review Board, and all participants provided written informed consent. Pursuant to the inductive nature of the qualitative approach, the study allowed focus to remain on participants’ experiences, bringing forth their preferences, needs, and understanding of what they saw as leading to better suited health care.^35^ The study team created the structure and content of interview questions, based on literature review, leading to creation of a predetermined and semi-structured sequence of questions. The specific domains of inquiry focused on barriers to treatment, mind–body intervention interest and acceptability, and feasibility of various intervention formats. The 4 mind–body techniques explored included diaphragmatic breathing, cognitive reframing, inner smile meditation, and guided visualization.

We had hoped to use a focus group method exclusively but adapted to add individual interviews based on the availability of participants and the ability to schedule them for a video conference call. AYA were in 1 focus group together, whereas parents each had individual interviews. Three of the providers completed individual interviews, and 3 were in a focus group together. Moderators facilitated interviews and focus groups, and a co-researcher recorded observations and monitored the recording of the sessions, making sure that audio and video recordings were valid. The semi-structured interviews encouraged participants to express their perceptions, minimizing limits set by standardized questionnaires, so as to minimize investigator-imposed bias, as well as to elicit valuable insights into personal and social phenomena.^36^ Duration of interviews ranged from 30 to 70 minutes, with an average length of 40 minutes.

Focus groups and individual interviews were transcribed verbatim. The transcripts were de-identified. Consensual Qualitative Research (CQR) is an inductive method used to look for themes and subthemes, allowing for the study of attitudes, beliefs, and personal inner experiences among participants.^37^ This method relies on small samples, open-ended questions, consensus among research team members, as well as integration of multiple perspectives of the research team, unlike other methods that mostly rely on a single interpretation of data.^37^, ^38^, ^39^ The suggested sample size is 8 to 15 participants.^38^

The auditor, a psychologist, is an experienced CQR researcher who received qualitative methods training through graduate education, completed dissertation work using qualitative methods (CQR), and participated in numerous qualitative research projects. The research team responsible for coding the data consisted of 2 psychiatrists and a clinical research specialist. All have had prior qualitative research experiences, studied available literature on CQR method, and followed CQR structured manuals.^37^^,^^38^ Research team members consulted with the auditor and senior qualitative method researcher, who is also a psychiatrist. The research team reviewed and coded each transcript independently, then met to review and discuss differences until consensus was reached, as suggested by the CQR analysis. Core ideas for each section were constructed to represent statements in a more succinct and clear manner. The auditor reviewed the consensus version of each transcript and provided feedback during each phase of coding. The purpose of auditing was to help decrease “group-think” and to maintain objectivity.^39^ Four final themes emerged from the data, followed by cross-analysis, which ultimately led to the creation of the final categories.

## Results

Analysis revealed 4 domains: Barriers to the Management of T2D, Facilitators to the Management of T2D, Intervention Content, and Intervention Format, as illustrated in Table 1. The following legend corresponds to the participants in our data below: F = facilitator; P = provider; A = AYA; and G = guardian.

**Table 1:** Domains and Categories of the INTEND Intervention Study

Domains and categories	Definitions
Barriers to the Management of T2D	Internal and external factors that interfered with self-care and management of the disease
Psychoemotional barriers	Psychological and emotional factors negatively affecting one’s ability to manage T2D
Social barriers	Community attributes that may negatively affect ability to manage T2D
Medical barriers	Perceived pitfalls within health care system negatively affecting management of T2D, and perceived differences in resources and treatment of T1D and T2D populations
Behavioral barriers	Behaviors and routines that may negatively affect management
Facilitators to the Management of T2D	Internal and external factors and habit/life changes that may aid in care while living with T2D
External support facilitators	Supportive community attributes
AYA behavioral facilitators	Activities and behaviors that positively affect management of T2D
Psychoemotional facilitators	Psychological and emotional factors that positively affect management of T2D
Intervention Content	Topics of interest for intervention sessions: favorable experiences and interventions
Mindfulness interventions	Proposed mindfulness interventions: diaphragmatic breathing, guided meditation, cognitive reframing, inner smile meditation
Basics of T2D	Demand for psychoeducation and basic mechanisms behind T2D management
Intervention organization and acceptability	Strategies to keep in mind while creating content to increase openness to participation
Intervention Format	Preferences and logistical information of proposed intervention series
Frequency	Preferences for frequency, duration, and quantity of sessions: 6-8 weekly sessions, session duration of 45 minutes
Mode	Virtual vs in-person intervention: virtual preferred and deemed more accessible
Facilitator attributes	Specific attributes of session leaders, facilitators, and presenters that may enhance engagement
Structure	Clear session structure, parental engagement, proactive check-ins, groups vs individual sessions, means of communication, incentives, and resources

AYA = adolescents and young adults; T1D = type 1 diabetes; T2D = type 2 diabetes.

### Barriers to the Management of T2D

This domain captured the internal and external factors that participants believed interfered with their self-care and management of the disease. It included the following 4 categories, highlighting the interaction of the disease mechanisms’ negative impact on one’s life, and vice versa.

1. Psychoemotional barriers (ie, the psychological and emotional factors negatively affecting the ability to manage T2D): Participants narrated feelings of isolation, anxiety, bullying, lack of understanding by peers not diagnosed with T2D, chronic disease fatigue, stigma, blame, perceived lack of control, feeling overwhelmed, guilt, depression, and lack of engagement and cooperation. One AYA participant expressed challenges with accepting a lifelong diagnosis, while acknowledging feelings of shame:

It's …the way that it is talked about, not just in the medical setting, but outside of your medical setting. It's very much something that you are made to feel ashamed for. Or it's your fault for [having it], when it's not the case. And even if you do lose weight, like I was told when I was diagnosed, even if you do lose weight, you'll be diabetic your entire life. The status won't change. [A3]

2. Social barriers: Communities in which patients reside may negatively affect their ability to manage their T2D through parental disengagement (eg, expecting teens to independently self-manage and potentially not providing emotional support or appropriate nutritional options), household structure and dynamics (eg, chaotic household), and limited community support. Racial divide and lack of trust in medicine were viewed as barriers to overall management. A provider offered a glimpse of the detriments set forth by environmental limitations:

...living in communities where you don't have…grocery stores or …fresh produce, ...when the corner store is the closest place and if you're hungry…, you're going to go to the corner store and get chips and a pop or a juice… healthy foods…is definitely a barrier too… or just access in general … to facilities to exercise …safe playgrounds, safe areas. [P1]

3. Medical barriers: Perceived pitfalls within the health care system negatively affecting the management of T2D, and perceived differences in resources and treatment of patients with T1D and T2D. Participants also focused on limited professional support, and the lack of communication with health care providers. A parent described their struggle with the lack of services and support offered:

...there's a huge divide between type 1 and type 2 kids, families, and services that can be accessed. …when she was first diagnosed we were offered a whole lot …like the Juvenile Diabetes Research Foundation… But the truth is that they're exclusive… to type 1. ...They had camps for teenagers with type 1. But not teenagers with type 2. [G1]

4. Behavioral barriers (eg, behaviors and routines that may negatively affect the management of T2D): One AYA participant verbalized consistently forgetting to take medicine coupled with self-deprecation as leading to challenges in her management of diabetes.

### Facilitators to the Management of T2D

This domain included internal and external factors and habit/life changes that may aid in care while living with T2D, including resources provided to patients and parents.

1. External support facilitators: Community attributes that support the management of T2D such as family support, professional support, diabetes camp, early intervention, positive role modeling, support groups, and access to superior technology. An AYA participant remarked how having family members who understand what she is going through can help manage the chronic disease:

One of the positives that I have noticed just because of the fact that it's kind of a genetic thing, like, both my parents had it and almost all of my grandparents had it. That in my family, we can, like, make [early dietary] switches that are going to help my younger sisters; …it might help them a little bit later on down the line, with kind of helping them keep better management of their sugar if they do get diagnosed with being diabetic. [A1]

2. AYA behavioral facilitators: Activities and behaviors that positively affect management of T2D appeared mostly in the form of good organizational skills, creating helpful routines, and attainable goal setting:

Instead of thinking, “oh yeah, I gotta drop 50 pounds”, no! Let's drop maybe 10 pounds…by just doing small things, maybe…cutting out sugary beverages, or cutting out…something…in a smaller scale. [P5]

3. Psychoemotional facilitators: Psychological and emotional factors that positively affect teens’ ability to manage T2D: instilling hope, positive body awareness related to the TD2 experience, a sense of being in control, and relevance of care to AYA priorities.

…I would see a positive that I like to look at is you know more about your body… You're more aware of how your body feels every day and like what your body is like when levels are at a normal basis. I have friend who has another best friend who is a type 1 diabetic. Dude doesn't know anything about diabetes like at all… But, me and our other friend who's a type 1 diabetic. We, the 2 of us, know like what our sugar should be at. And how do we feel when this happens? … He could be hungry and shaking and stuff, and he'd be like I don't know I might just be sick today. Well, the 2 of us are like, ‘Is your sugar okay? Are you okay today?’ And that's, I think, that's one of the positives about having type 2 diabetes. That you know what's going on with your body on a regular basis. [A1]

### Intervention Content

The intervention content domain focused on topics of interest for intervention sessions, as well as favorable experiences and interventions to include. It consisted of 3 categories.

1. Mindfulness interventions: Diaphragmatic breathing, guided mediation, cognitive reframing, inner smile meditation, and appropriate role of these techniques. Mindfulness interventions discussed were deemed acceptable and helpful by participants in this study.

I think it’s a great idea to try [guided visualization]... meditation is very popular now, and …friends and family members who use it …get a lot of benefit from it. I don’t really have a lot of experience with it, but... I've been through some here and there learning about it, but I would say…encouraging the kids to try it more than once…not giving up …feeling like, “oh this is dumb” or “ I can't stop thinking about what I'm thinking about” but…reinforcing...this might take a little bit of training your mind. [P4]

2. Basics of T2D:The need for psychoeducation and basic mechanisms behind T2D management, and evidence in support of treatment options, such as nutrition, and scientific support.

…the person who's coaching them being, you know, very warm and sensitive… somebody who can explain it well, and the potential benefits of it. [G2]

…make sure we give them the “why” as to what we're doing here... [F]

…explaining …the benefits that the research shows. [G2]

3. Intervention Organization and Acceptability: Strategies to keep in mind while creating content, to increase openness to participation and incorporate the recommended interventions into daily routine. These included personalizing sessions, support for parents, and a supportive approach toward the AYA with chronic illness:

Having a structure of, “here's 3 things [to do] when that happens. Here's 3 things when this happens. Here’s 3 things when that happens.” You wanna get mad, here's, “go downstairs and [hit] the punching bag.” [P1]

To help her work through and pick some, a bunch of different [ways to cope]… that makes lots of sense. [F]

… nothing's gonna work all the time, 100% ... you need a lot of different ways to cope. [P1]

### Intervention Format

Intervention format highlights preferences and logistical information regarding the proposed intervention series (time, location, duration, attendees), with the ultimate goal of enhancing delivery.

1. Frequency: Preferences for frequency, duration, and quantity of sessions were explored. Participants favored 6 to 8 weekly sessions, with the average session duration of 45 minutes. Participants underscored elements that can enhance participation, such as practice encouragement, flexibility of session schedule, and timing of sessions.
2. Mode: A preference for virtual intervention was evident. In-person sessions, although highly valued, may present barriers to access. Virtual engagement was deemed as more accessible, with a possibility of optional in-person sessions. Participants valued access to informational materials during and after sessions through an app or a printed document.
3. Facilitator Attributes: The significance of specific attributes of session facilitators that may enhance engagement: caring, trustworthiness, having a peer educator, relatability, and flexible teaching styles. A provider verbalized the significance of matching facilitators:

…I don’t know how much [it’s] the packaging of the person, but do the kids feel like you’re connected to them and you’re really invested in them…[F1]…Yeah…I think if you have a dynamic doctor, nurse, social worker, whatever. [P1]

4. Structure**:** Clear session structure, parental engagement, proactive check-ins between sessions, group vs individual sessions, means of communication with participants, incentives to participate, and resources were all deemed significant. Depending on program activities, parental engagement and support were deemed valuable.

…parents [and kids together is] complicated. They’re all, obviously different—different goals, different personalities… parents and kids separate I think helps a lot. …when they're in front of their kid a lot of times… they're worried about being judged, so they blame the kid. “Oh Jimmy sneaking food, is doing this, doing that.” Pull the mom or dad out, and then get an understanding of what is going on in this house with certainty…. Because parents …learn a lot about why they're not supportive. …sometimes they're at their wits end. They don't have a lot of support...they're stressed about basic needs…[P4]

## Discussion

To help craft an intervention for youth with T2D that encompasses mind–body interventions (a.k.a., INTEND), we conducted several focus groups of individuals who have personal experience with T2D from the patient, parent, and caregiver perspectives. Qualitative research methods revealed perspectives on priorities for the intervention content and format, and explored the acceptability of 4 specific mind–body interventions. The results of our qualitative analysis identified a number of barriers to self-management that have important implications for how best to support and empower this high-risk group. The most salient barriers to T2D management appear to be psychosocial factors. Perceived lack of understanding by peers not diagnosed with T2D, bullying by peers and significant others, and feeling blamed by others were recognized as significant barriers. This outcome is of the utmost importance, as adolescence is a developmental period for establishing autonomy, identity, and patterns of peer relationships.^40^ The vast differences in compassion received and resource allocation experienced by patients with T2D in comparison to patients with T1D stood out as well. T2D-related social stigma in adults has been well documented,^41^ and may originate in misconceptions that individuals with T2D caused their state of ill health by poor dietary choices, obesity, and laziness.^42^ This type of diabetes-related social stigma has not been well studied in AYA. Our study found evidence of perceived social stigma related to AYA with T2D that appears to mirror that in adults. Participants in this study believed that the unequal management of patients with T1D and T2D, as well as the stigma of T2D as a lifestyle disease among peers, were significant barriers to being able to manage their T2D.

On an intra-personal level, AYA perceived lack of control and feeling overwhelmed as barriers to effective self-management. Parent and provider groups considered insulin management and administration, chronic disease fatigue, and parental disengagement to be significant barriers to the youth being able to manage their T2D. Depression was also recognized as a significant contributor to management challenges, aligning with research reports that have called for a better understanding of youth with T2D and depressive symptoms.^7^^,^^13^^,^^19^ Our results also indicate that this AYA population is at an increased risk for experiencing diminished emotional well-being and physical health.^12^^,^^13^^,^^43^

Very little is known about youth-onset T2D management facilitators, which can ease the management of chronic disease. Youth, parents, and providers in this study highlighted various factors that may improve disease management and self-care. Participants identified key facilitators needed as a component of any intervention, including INTEND, which involved making the intervention relevant to AYA priorities, providing professional support to AYA and their families, helping to teach ways to manage stress and perceived isolation, and ensuring that educators were relatable to the AYA population. Support and understanding by family and peers added to better outcomes of T2D management overall in this group.

We identified several aspects of the mind–body intervention format that may increase AYA acceptability and adherence. These included consistency of intervention format, including frequency and duration of group sessions. Interventions delivered electronically appear to be the most feasible and preferred format, particularly from the AYA perspective. Somewhat in contrast, parents and providers preferred in-person interventions. However, they noted that AYA may be more receptive to an online format. In addition, the online format offers easier access, and removes many barriers to attendance, such as the cost of gas and parking, and time invested to get to the session. Hybrid approaches may be needed to be most effective for both AYA and parent groups.

The preferred length/duration of programing was 6 to 8 weeks, with an average of 45-minute sessions. Participants suggested that having access to all presented tools after the program is over would be preferable, whether in a paper or an electronic format. The qualities and attributes of the group facilitator were noted to be critical. Participants preferred someone relatable to the AYA population, such as a peer with similar experience, in addition to 1 or more professionals who are credibly authentic.

Other factors deemed significant toward the satisfaction and approval of the proposed interventions included the following: support groups for AYA and parents with an emphasis on living with chronic illness; conveying the appropriate role in AYA daily life of the mind–body techniques taught; scientific support and explanation of the etiology of T2D; avoiding scare tactics; as well as the importance of including a nutritional component with assistance from a dietician. Additional facilitators included a dedicated, AYA-only group, and then adding combined time with parents, when appropriate.

Data revealed multifaceted barriers and facilitators to T2D management that AYA face on a daily basis and that inform the development of interventions toward improving their experience and outcomes. Mind–body techniques were both acceptable and desired by AYA and their families, as well as by medical providers working with this population. This type of exploration rarely appears in the literature for this specific population, yet this population has unique needs that must be accounted for and addressed for interventions to be holistically applicable. The authors hope that this overview of barriers and facilitators in AYA with T2D can inform an optimal mind–body intervention that is personalized, feasible, and easy to integrate and maintain in daily life, and can inform others working to best serve this cohort. Our results suggest that mind–body interventions, especially those delivered remotely or via smartphone applications, may be a favorable addition to care in this population. Further research of this type—engaging larger and more diverse populations of AYA with T2D—are needed to more thoroughly capture their unique needs, and to identify needs that may be present in groups from different cultures, subcultures, ethnicities, and socioeconomic backgrounds. It is important to note that even in this small sample, some barriers and facilitators were not uniform, underscoring the need to personalize intervention approaches to enhance adherence and use. The INTEND study is the first effort, to our knowledge, that has developed an intervention model (the INTEND intervention) based on qualitative research involving youth with T2D and their providers and caregivers.

Our study engaged a small sample from a single urban setting in the United States, which may limit transferability of the findings to other populations in other locales. AYA were all female participants, which may limit information on perceived facilitators and barriers for a study incorporating mind–body interventions. Future iterations of INTEND study may illuminate this information further by separating the sample into 3 distinct groups (youth, parent, and provider) to gain more knowledge regarding this population. Each of these unique perspectives provided meaningful and indispensable information. We will aim to include more diverse populations, to increase numbers of participants, and to improve our recruitment strategy in future iterations of this work. Future research into INTEND as an add-on to treatment, especially when scaling to a larger population, will need to address cost and barriers to large-scale implementation.
